# Biodegradation of ZEN by *Bacillus mojavensis* L-4: analysis of degradation conditions, products, degrading enzymes, and whole-genome sequencing, and its application in semi-solid-state fermentation of contaminated cornmeal

**DOI:** 10.3389/fmicb.2025.1512781

**Published:** 2025-03-26

**Authors:** Jiagao Chang, Wenxuan Dong, Shansong Gao, Lele Hou, Jihong Dong, Huiling Qiu, Fu Chen

**Affiliations:** ^1^Institute of Animal Nutritional Metabolic and Poisoning Diseases, College of Veterinary Medicine, Qingdao Agricultural University, Qingdao, Shandong Province, China; ^2^Haidu College, Qingdao Agricultural University, Laiyang, Shandong Province, China

**Keywords:** zearalenone, *Bacillus mojavensis* L-4, biodegradation, degradation products, peroxidase efeB 3,668

## Abstract

Zearalenone (ZEN), a naturally occurring estrogenic mycotoxin prevalent in cereals and animal feed, poses significant challenge to livestock industry owing to its detrimental effects on animal reproduction. In this study, the strains with high degradation rate were screened through co-culture with ZEN, and identified by bacterial morphology, 16S rDNA sequencing and whole genome sequencing. The detoxification effect of L-4 strain on ZEN was evaluated under different ZEN concentration, treatment time, pH value and temperature, the degradation products were identified, and the degradation effect of L-4 strain on ZEN contaminated corn meal was evaluated. The ZEN degrading enzyme sequence was obtained through the whole genome protein sequence analysis of strain L-4, and the ZEN degrading enzyme was verified by molecular binding and addition of catalase. We isolated *Bacillus mojavensis* L-4 from the cecal content of laying hens, which demonstrated exceptional ZEN-degrading efficiency. Under optimized conditions (pH 7.0, 37 °C), L-4 completely degraded 0.5–1.0 μg/mL ZEN into less toxic 15-OH-ZEN within 24 h. Importantly, L-4 achieved a 49.41% degradation rate for ZEN in cornmeal. Whole-genome sequencing of L-4 revealed the presence of ZEN-degrading genes and enzymes. In particular, efeB 3668, a peroxidase-like enzyme with high homology (95.91%) to BsDyP from *Bacillus subtilis*, played a key role in ZEN detoxification primarily through hydrogen bonding and hydrophobic interactions. Thus, the rapid and effective degradation of ZEN by *B. mojavensis* L-4, coupled with its adaptability to diverse environments, underscores its potential application in safeguarding animal health and mitigating environmental pollution.

## 1 Introduction

Zearalenone (ZEN), commonly known as F-2 toxin, is a non-steroidal estrogenic mycotoxin produced by various *Fusarium* and *Gibberella* spp. It is one of the most pervasive contaminants found in grains and animal feed, posing a formidable challenge to food safety and livestock health (Han et al., 2022). Owing to its structural similarity to the endogenous hormone 17β-estradiol, ZEN exhibits potent reproductive toxicity, capable of disrupting the endocrine balance in animals, inducing hormonal imbalances, and adversely affecting growth and reproductive performance (Zheng et al., 2018). There is an alarming increase in ZEN contamination in agricultural products and feedstocks, with detection rates as high as 45% across Europe, America, and Asia and an astonishing 96.9% in Chinese feed samples (Gruber-Dorninger et al., 2019; Zhao et al., 2021), thus highlighting the urgent need for developing efficient strategies to mitigate the toxic effects of ZEN.

One of the primary challenges in ZEN management arises from its remarkable thermal stability, allowing it to persist even at temperatures as high as 220°C for an hour (Murtaza et al., 2022). Consequently, conventional thermal processing methods are ineffective in eliminating ZEN. Current ZEN detoxification approaches encompass physical, chemical, and biological methods, each with their own limitations. Physical adsorption and chemical detoxification techniques often exhibit low efficiency, can potentially compromise the nutritional value and palatability of feeds, and can accidentally introduce harmful compounds (Shanakhat et al., 2018; Zheng et al., 2018; Yang et al., 2020). In contrast, biological methods exploit the innate decomposing abilities of microorganisms, offering a highly efficient, specific, and environmental-friendly alternative for ZEN degradation. By disrupting the toxic moieties within the ZEN molecule, these microorganisms or their enzymes can convert ZEN into non-toxic or less toxic compounds (Ji et al., 2022). However, it is crucial to note that ZEN degradation may not always lead to complete detoxification, because it can sometimes yield estrogenic analogs such as *α*-zearalenol and *β*-zearalenol, which retain toxic properties (Zhang et al., 2020). Numerous bacteria and fungi, including *Bacillus* spp., *Lactobacillus plantarum*, and *Saccharomyces cerevisiae*, along with their associated enzymes, have demonstrated the ability to transform ZEN into less harmful products (Gao et al., 2017; Medeiros et al., 2018; Chlebicz and Śliżewska, 2020; Møller et al., 2021). For example, Chlebicz and Śliżewska (2020) reported good ZEN adsorptive capacity of *S. cerevisiae*, while Møller et al. (2021) isolated a *L. plantarum* strain from Brazilian cheese with robust ZEN degradation capabilities.

In the present study, a novel *Bacillus mojavensis* strain with remarkable ZEN-degrading capabilities was isolated. Subsequently, comprehensive investigation of the optimal degradation conditions, enzymes involved, and products generated from ZEN degradation by this novel strain was performed. A ZEN-degrading peroxidase, designated as efeB 3,668, was identified in *B. mojavensis* L-4, and its kinetic properties and crucial amino acid residues were further examined. Mass spectrometry analysis of the degradation products confirmed that *B. mojavensis* L-4 and its secreted enzyme efeB 3,668 could successfully convert ZEN into 15-OH-ZEN, a derivative with significantly reduced estrogenic toxicity.

Specifically, our study advances previous research in the following ways: Unique Strain Identification: We identified and characterized a novel strain, L-4, which exhibits superior ZEN degradation efficiency compared to previously reported strains. The content of ZEN was measured using HPLC, and it was found that 0.5 to 1.0 μg/mL of ZEN in liquid medium could be completely degraded at 24 h.

Mechanistic Insights: Unlike prior studies that primarily focused on ZEN degradation pathways, our work provides new insights into the enzymatic mechanisms, particularly the role of Dyp-peroxidase, in ZEN degradation. After identification, prediction and molecular binding, we found that efeB3668 has a high homology with the peroxidase BsDyP, and there is a good interaction between the peroxidase and ZEN, which fully indicates that the peroxidase can effectively degrade ZEN. However, the ability of efeB3668 to degrade ZEN needs further exploration.

Practical Application: Previous studies have often only focused on the degradation efficiency of degrading bacteria in liquid media. We demonstrated the potential of strain L-4 for practical application in reducing ZEN contamination in cornmeal, offering a promising solution for improving food safety.

These findings provided a solid foundation for exploring innovative ZEN biodegradation technologies, offering promising avenues for mitigating the detrimental effects of this persistent mycotoxin.

## 2 Materials and methods

### 2.1 Chemicals, media, and test strains

ZEN (purity ≥99.4%) was procured from Qingdao PureBio Bioengineering Co., Ltd. HPLC-grade methanol and acetonitrile were obtained from Tianjin Kemiou Chemical Reagent Co., Ltd. The bacterial growth and ZEN degradation medium consisted of 15.0 g/L peptone, 5.0 g/L yeast extract, 2.0 g/L potassium dihydrogen phosphate, 10.0 g/L glucose, 2.5 g/L tomato extract powder, and 1.0 g/L Tween 80, with pH adjusted to 7.0. The fungal growth and ZEN degradation medium contained 20.0 g/L yeast extract and 20.0 g/L peptone, with a pH of 6.5. For obtaining solid medium, agar (12.0 g/L) was added to the liquid medium and sterilized at 121°C and 0.1 MPa for 20 min in an autoclave (Shanghai Boxun Industrial Co., Ltd., China). Template DNA, upstream primers, downstream primers, 10 × PCR Buffer, dNTP, Taq Plus DNA Polymerase, MgCl_2_, catalase (2000 IU) and ddH_2_O was purchased from Sangon BioEngineering (Shanghai) Co., LTD. All strains used in this study were isolated and preserved in the Laboratory of Animal Nutrition, Metabolism, and Toxicology, Qingdao Agricultural University, with the following codes: E-1 (*Enterococcus faecalis*), L-2 (*Lactobacillus rhamnosus*), L-3 (*Lactobacillus delbrueckii subsp. bulgaricus*), L-4 (*Bacillus mojavensis*), L-5 (*Lactobacillus plantarum*), L-6 (*Lactobacillus parasei*), B-7 (*Streptococcus thermophilus*), B-8 (*Bacillus licheniformis*), B-9 (*Bacillus cereus*), S-10 (*Saccharomyces cerevisiae*), B-11 (*Bacillus subtilis*).

Animal ethics statement: The L-4 strain used in the experiment was isolated from the cecal contents of healthy Hy-line brown laying hens by previous researchers in our laboratory. The animal experiments were approved by the Qingdao Agricultural University Animal Care and Use Committee (Qingdao, China) in accordance with Laboratory Animal Guidelines for the Ethical Review of Animal Welfare (GB/T35892-2018, National Standards of the People’s Republic of China).

### 2.2 Preliminary evaluation of ZEN-degrading strains

Eleven candidate strains were inoculated in liquid medium for resuscitation. In this experiment, 3.8 mL liquid medium and 200 μL fermentation bacteria solution were added to a 10 mL sterile enzyme-free centrifuge tube to control the ZEN concentration at 0.5 μg/mL. The centrifuge tubes for ZEN degradation by 10 bacterial strains were incubated at 37°C and 160 rpm for 48 h, respectively. In the control group, only the medium containing ZEN was added. The centrifuge tubes for ZEN degradation by one strain of fungi were incubated at 30°C and 160 rpm for 72 h. The control group contained only ZEN without any strain. After incubation, 460 μL of the samples were mixed with 460 μL of acetonitrile and 80 μL of methanol in sterile centrifuge tubes, vortexed for 5 min, and filtered through a 0.22-μm PTFE membrane. The presence of ZEN in the cultures was detected using HPLC with a UV detector (LC-20A, SHIMADZU CORPORATION, Japan) at 274 nm (Xu et al., 2020). Separation was performed on a C18 column (150 mm × 4.6 mm, 5 μm, Yuexu Technology Shanghai Co., Ltd., China) with an injection volume of 20 μL and a column temperature of 30°C. The mobile phase consisted of acetonitrile-water–methanol (46: 46:8, V/V/V) at a flow rate of 0.8 mL/min, with ZEN eluted using 100% methanol for 5 min. A standard curve was generated based on the retention time and peak area of the ZEN standard stock solution to determine ZEN residues. All samples were performed three times, and the average values were then calculated. Strain L-4, which exhibited the highest ZEN degradation rate, was selected for further study and stored at −80°C in liquid medium containing 40% glycerol.

### 2.3 Morphological identification of ZEN-degrading bacterium

These strains were inoculated onto solid medium by the three-zone streaking method and incubated at 37°C for 24 h. Solid medium includes bacterial solid medium formula (peptone 15.0 g, yeast extract powder 5.0 g, potassium dihydrogen phosphate 2.0 g, glucose 10.0 g, tomato extract powder 2.5 g, Tween 80 1.0 g, Agar 12.0 g) and fungal solid medium formula (peptone 10.0 g, glucose 20.0 g, yeast extract powder 5.0 g, Agar 14.0 g). Subsequently, the color, size, shape, texture, transparency, and edge elevation of individual colonies were recorded. Then, a well-grown single colony was subjected to Gram staining and examined under a microscope (CK40-SL, Olympus, Japan) to observe the cellular morphology.

### 2.4 The 16S rDNA identification

DNA was extracted from strain L-4 using a DNA extraction kit, and PCR amplification of the 16S rDNA region was performed using primers 27F (5´-AGAGTTTGATCMTGGCTCAG-3′) and 1492R (5´-GGTTACCTTGTTACGACTT-3′), as described by Wang et al. (2019) The PCR reaction system consisted of 1.0 μL template DNA, 1.0 μL 27F, 1.0 μL 1492R, 0.5 μL Taq DNA Polymerase, 0.5 μL dNTP, 2.0 μL MgCl_2_, 2.5 μL 10 × PCR Buffer, and 16.5 μL ddH_2_O. The conditions were predenatured at 94°C for 5 min. 35 cycles of 94°C for 30 s, 60°C for 30 s, and 72°C for 1 min were followed by an extension of 8 min. Among them, the concentration of DNA was 50 ng/μL, and the working concentration of forward and reverse primers was 10 uM. The PCR products were verified by 1.2% agarose gel electrophoresis at 120 V for 40 min. The positive bands were sequenced by Shanghai Sangon Biotech Co., Ltd. (Shanghai, China), and the 16S rDNA sequence of strain L-4 was submitted to the National Center for Biotechnology Information (NCBI) with GenBank ID OR 649301.1. The closest related sequences were retrieved from the NCBI database and a phylogenetic tree was constructed using the maximum likelihood method by comparing homology with selected Bacillus model strains by utilizing MEGA 5.10 software.

### 2.5 ZEN degradation efficiency under different cultivation conditions

To investigate the degradation rate of ZEN by L-4 strain under different conditions, 1 mL of fermentation bacteria solution was added to a conical flask containing 19 mL of liquid medium, shaken at 240 rpm, and samples were collected at regular intervals. HPLC was used to quantify the amount of ZEN remaining, and the degradation rate was calculated to evaluate the degradation of ZEN by the L-4 strain. Culture conditions included time (3 h, 6 h, 9 h, 12 h, 15 h, 18 h, and 21 h), temperature (25°C, 37°C, 40°C, 42°C, and 45°C), initial pH (2.5, 4.0, 5.0, 6.0, 7.0, 8.0, 9.0, 10.0, and 11.0) and different initial ZEN concentrations (0.5 μg/mL, 1.0 μg/mL, and 5.0 μg/mL). A control group was set up, and only ZEN standards were added to the liquid medium. All samples were performed three times, and the average values were then calculated.

### 2.6 Evaluation of ZEN degradation by different components of strain L-4

The ZEN degradation efficacies of different components of strain L-4, including viable bacteria, heat-inactivated bacteria, bacterial intracellular fluid, bacterial debris, bacteria-free supernatant, heat-inactivated bacteria-free supernatant, and bacteria-free supernatant treated with proteinase K/SDS, were assessed. The bacterial cultures were centrifuged at 8,000 rpm for 20 min to obtain cell-free supernatants, and the bacterial precipitates were resuspended in PBS to obtain live and heat-inactivated bacterial suspensions. Intracellular fluid was extracted from the suspension of live bacteria subjected to ultrasonic fragmentation and the fragments were resuspended in PBS. All the treatment groups were mixed with 0.5 μg/mL ZEN, whereas the control comprised PBS or liquid medium containing ZEN. All the samples were incubated at 37°C and 240 rpm for 72 h. After centrifugation and filtration, the ZEN residues were detected by HPLC.

To determine whether ZEN degradation was enzyme-dependent, cell-free supernatants were heat-treated at 100°C for 10 min. Then, SDS (final concentration of 1%), proteinase K (1 mg/mL), and a combination of proteinase K + SDS were added to the samples and incubated for 72 h, and the samples were analyzed by HPLC to evaluate ZEN degradation.

### 2.7 Analysis of ZEN degradation products

In order to observe the final phase of ZEN presence, we chose to sample the reaction at 20 h for subsequent analysis of degradation products by UPLC-Q-Exactive-MS/MS. The strain L-4 and ZEN were added to the liquid medium and incubated at 37°C and 240 rpm for 20 h. The reaction was terminated by adding an equal volume of methanol, followed by vortex mixing for 10 min. The mixture was filtered through a 0.22-μm PTFE membrane into a liquid chromatography vial. The control group consisted of liquid medium and ZEN. Identification and analysis of the samples were performed using UPLC-Q-Exactive-MS/MS (Thermo Fisher Scientific, United States).

Chromatographic Conditions: An Agilent Poroshell 120 EC-C18 column (2.1 mm × 100 mm, 2.7 μm) was employed, with a column temperature of 30°C, an injection volume of 5 μL, and a flow rate of 0.2 mL/min. The mobile phases consisted of methanol (A) and water containing 0.1% formic acid and 5 mmol/L ammonium formate (B). The gradient elution program was set as follows: 0–0.5 min, 90% B and 10% A; 0.51–5 min, 90–5% B and 10–95% A; 5.01–8 min, 5% B and 95% A; and 8.01–9 min, 90% B and 10% A.

Mass Spectrometry Conditions: An electrospray ionization source was used in both positive and negative ion scanning modes with multiple reaction monitoring. The ion source temperature, drying gas flow rate, nebulizer gas pressure, capillary voltage, and scan range were set at 350°C, 8 L/min, 25 psi, 3,000 V, and m/z 50–1,000, respectively. For MS/MS analysis, the induced collision dissociation energy was set at 20 eV.

### 2.8 Degradation of ZEN in cornmeal by strain L-4

To investigate the effectiveness of strain L-4 in degrading ZEN in corn products, a degradation experiment was conducted using cornmeal. In this experiment, to ensure that the added bacterial solution was a single variable. A total of 10.0 ± 0.01 g of finely ground cornmeal were added to a 250-mL Erlenmeyer flask and sterilized in an autoclave at 121°C and 0.1 MPa for 20 min. Subsequently, ZEN standard was added under aseptic conditions to achieve a final concentration of 532.91 μg/kg, and the flasks were inoculated with 10% L-4 bacterial suspension (1.5 × 10^9^ cfu/mL). The control group only contained 10% sterile liquid medium. Each treatment comprised three replicates. All the flasks were incubated at 37°C for 5 days, and samples were collected at 0, 1, 2, 3, 4, and 5 days, respectively. The residual ZEN content in the cornmeal was detected using the up-converting phosphor technology-based lateral flow assay (Zhu et al., 2021), and the degradation rate was calculated as follows: Degradation rate = [(ZEN content in control group - ZEN content in experimental group)/ZEN content in control group] × 100%.

### 2.9 Whole-genome sequencing, degradative enzyme prediction, and molecular docking

#### 2.9.1 Whole-genome sequencing of strain L-4

The genomic DNA of strain L-4 was extracted using a bacterial DNA extraction kit (Omega, USA), and its concentration and purity were determined with a Thermo Scientific NanoDrop 2000 microspectrophotometer. Genome sequencing of strain L-4 was performed on the PacBio Sequel II platform (Shanghai Yuanxin Biomedicine Technology Co., Ltd., China). Filtered subreads were assembled using Canu v1.6 software, and the assembly was further corrected with Racon v1.4.20 utilizing third-generation subreads. Gene prediction was conducted with Prodigal v2.6.3 and Island Path-DIMOB v1.0.(She et al., 2009) The predicted gene-encoded proteins were compared with Clusters of Orthologous Groups of proteins (COG) and Kyoto Encyclopedia of Genes and Genomes (KEGG) databases in BLASTP (BLAST 2.2.28+) and reported with functional annotations. A genome circle plot of strain L-4 was generated using Circos software (Krzywinski et al., 2009). The mechanisms underlying ZEN degradation were explored from aspects such as cell motility and degradation-related gene prediction in strain L-4.

#### 2.9.2 Degradative enzyme prediction and molecular docking

The complete protein sequences obtained by sequencing were aligned with the six known ZEN degradation protein sequences in NCBI, and the sequence with the highest homology was identified using DNAMAN software. NCBI accession numbers and details for these sequences are as follows: Esterase ZHD101 (Clonostachys rosea IFO 7063, Accession Number: ALI16790.1), Phosphoric acid transferase ZPH (*Bacillus subtilis* Y816, Accession Number: QVL29807.1), Laccase CotA (*Bacillus licheniformis*, Accession Number: QAX90317.1), Peroxidase BsDyP (*Bacillus subtilis*, Accession Number: 7PKX_A), ZENC (Neurospora crassa, Accession Number: 1547034726), and ZLHY-6 (Pichia pastoris GSZ, Accession Number: QHW12260.1).

The subcellular localization of efeB 3,668 was predicted using PSORT server (Lau et al., 2021), and its hydrophobicity was assessed using ProtScale. SignalP 6.0 and TMHMM 2.0 servers were utilized to predict the presence of signal peptides and transmembrane regions in the amino acid sequence of efeB 3,668, respectively (Almagro Armenteros et al., 2019). The secondary structure of efeB 3,668 was predicted by SOMPA, and functional sites were analyzed using PROSITE and NCBI conserved domain search tools. Protein tertiary structure homology modeling of efeB 3,668 was conducted on the SWISS-MODEL web server using 7pkx.1.A as a template, and the reliability of the model was evaluated by the Ramachandran Plot server.

The 2D structures of small-molecule ligands were retrieved from the PubChem database,^1^ ZEN database link to https://pubchem.ncbi.nlm.nih.gov/compound/5281576 and converted into 3D structures in Chem Office 20.0 software (CambridgeSoft, United States). The docking receptor is the 3D structure of efeB 3,668 derived from homology modeling. Water molecules and phosphate groups were removed from the proteins using PyMOL 2.6.0 (DeLano Scientific LLC, United States; El-Hachem et al., 2017). Compounds were energy-minimized, and target proteins were preprocessed to identify active pockets using Molecular Operating Environment 2019 software (Chemical Computing Group Inc., Canada). Molecular docking was performed with MOE 2019, with 50 runs set for the calculation. Binding affinity was evaluated to assess the binding activity, and the results were visualized using PyMOL 2.6.0 and Discovery Studio 2019 (BIOVIA, United States).

#### 2.9.3 Validation of the major ZEN degrading enzymes

In order to test that the main degradation component of ZEN by the fermentation supernatant of strain L-4 was peroxidase, we added 10 μg/mL catalase to the culture supernatant of strain L-4 to remove H_2_O_2_ generated during the reaction, and sampled the remaining ZEN after 72 h to measure the degradation rate according to the method as described in section 2.8.

### 2.10 Data statistics and analysis

Statistical analysis was conducted using Microsoft Excel 2019 (Microsoft, United States) and SPSS 22.0 (IBM, United States; Dong et al., 2024). One-way ANOVA was employed for comparisons, and multiple comparisons of means were performed using the least significant difference test. The results are presented as the “mean ± standard deviation (SD), “with statistical significance indicated by *p* < 0.05. The phylogenetic tree of strain L-4 was constructed using MEGA 5.10. Mass spectrometry analysis of the degradation products was performed with Qualitative Analysis B.05.00 software (Agilent Technologies, United States). Degradative enzyme sequences were predicted using DNAMAN software (Lynnon Biosoft, United States), and graphs were generated with GraphPad Prism 9 (GraphPad Software, United States).

## 3 Results

### 3.1 Screening and identification of ZEN-degrading bacteria

**Figure 1:**
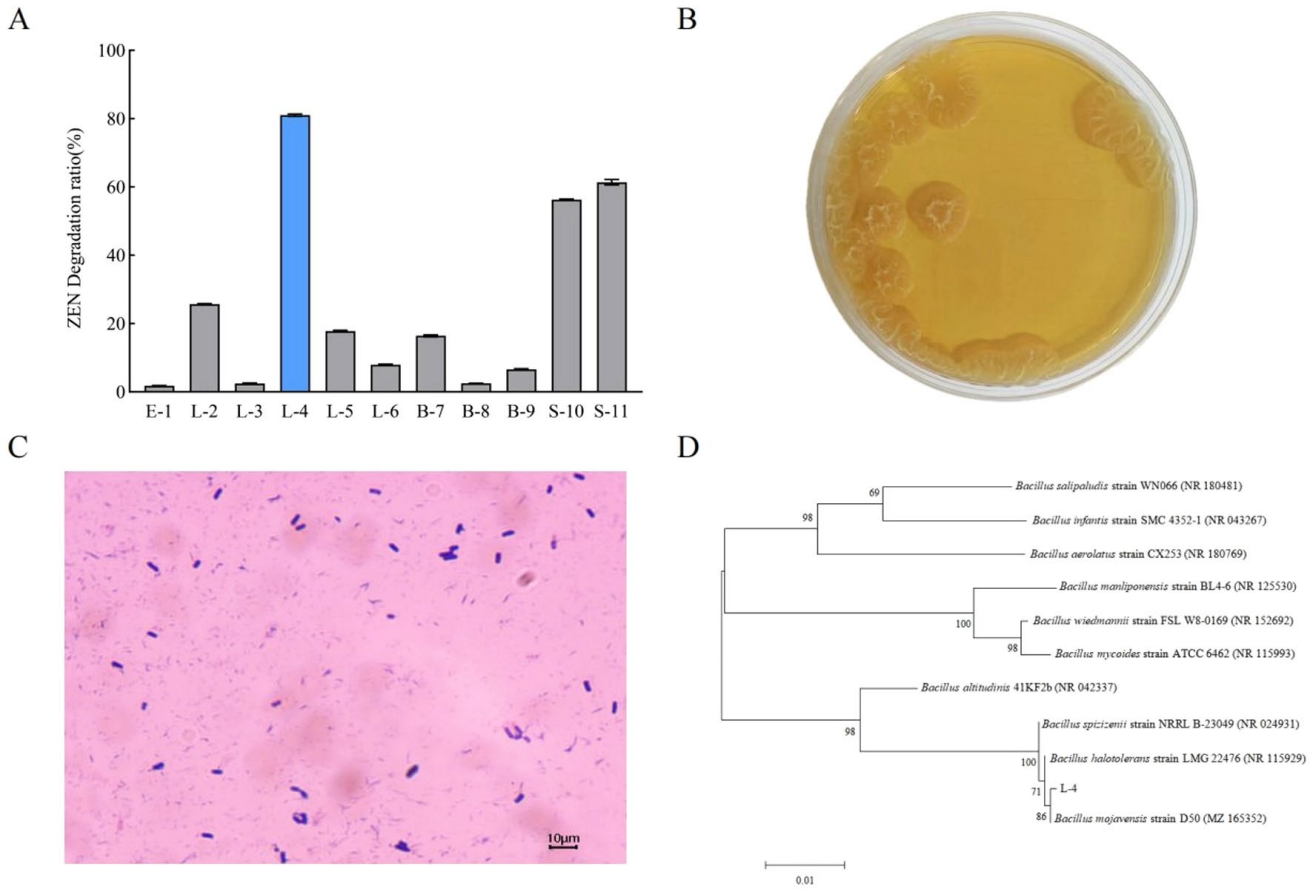
**(A)** Screening of ZEN-degrading bacteria. **(B)** Colony morphology and **(C)** cell morphology of strain L-4. **(D)** Phylogenetic tree of strain L-4 based on 16S rDNA gene sequences. Data are mean ± SD of three independent replicates.

As shown in Figure 1A, among the 11 strains, L-4, S-10, and S-11 presented better ZEN-degrading ability. In particular, strain L-4 exhibited the highest ZEN degradation rate of 81.04%, which was further investigated. As illustrated in Figure 1B, the colonies of strain L-4 were yellowish, opaque, rough, and wrinkled. Gram-staining further confirmed that strain L-4 was Gram-positive with rod-like morphology (Figure 1C).

Sequence homology analysis revealed a 99.65% similarity between strain L-4 and *B. mojavensis* D50 (GenBank ID: MZ165352.1) in the NCBI database, positioning strain L-4 within the same clade as *B. mojavensis* D50. Based on both colony morphology and 16S rDNA sequence analysis, strain L-4 was identified as *B. mojavensis*. The sequence obtained by 16S rDNA sequencing was submitted to GenBank and the accession number was OR 649301.1.

### 3.2 Degradation of ZEN by strain L-4

**Figure 2:**
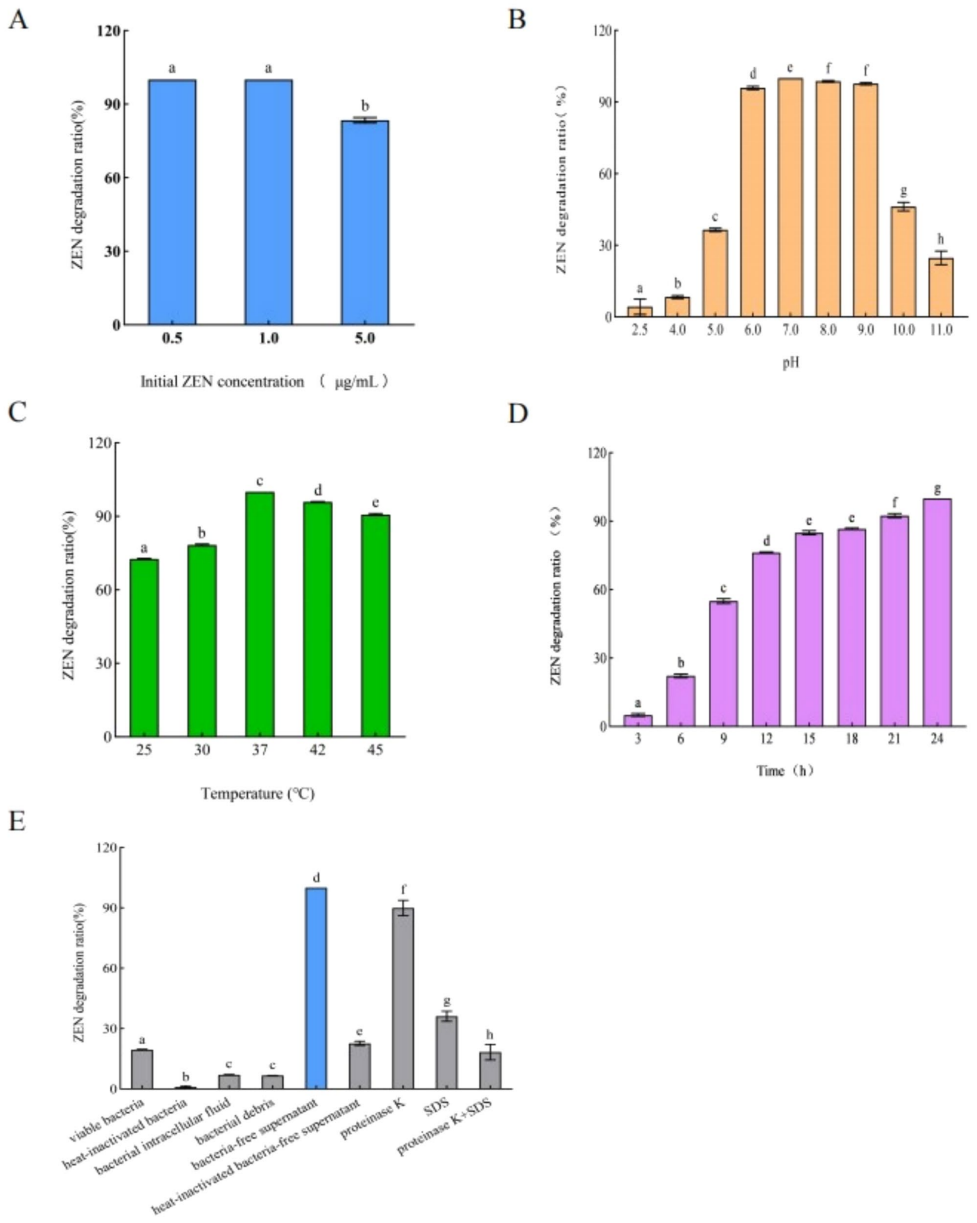
Effects of **(A)** initial concentration of ZEN, **(B)** pH of liquid medium, **(C)** temperature, and **(D)** time on the ZEN degradation rate. **(E)** ZEN degradation rates of different components of strain L-4. Data are the mean ± SD of three independent replicates.

As shown in Figure 2, the ZEN degradation rate of *B. mojavensis* L-4 was that after 24 h of incubation, ZEN was completely degraded in liquid medium. By optimizing pH, temperature and initial ZEN concentration, it was found that 0.5 and 1.0 μg/mL ZEN could be completely degraded after 24 h at pH 7 and 37°C.

### 3.3 Degradation of ZEN by different components of strain L-4

ZEN degradation rates of each components of strain L-4, including live bacteria, heat-inactivated bacteria, bacterial intracellular fluid, bacterial debris, and bacteria-free supernatant, were shown in Figure 2E. The ZEN degradation rate of sterile supernatant was the highest, reaching 100% at 48 h, while the ZEN degradation rates of bacterial intracellular fluid and live bacteria were 7.06 and 19.50%, respectively (Figure 2E). After inactivating the sterile supernatant and adding protease K (1 mg/mL), SDS (final concentration 1%) or SDS + protease K, the ZEN degradation rates declined to 22.69, 89.88, 36.13, and 18.13%, respectively (Figure 2E). The degradation rate of ZEN was significantly reduced after treatment of bacteria-free supernatant with protease K, SDS, protease K + SDS, and 100°C heat treatment, which strongly suggested that strain L-4 produced an extracellular enzyme responsible for ZEN degradation. However, further research is needed to fully understand the specific degradation mechanism.

### 3.4 Analysis of ZEN degradation products

**Figure 3:**
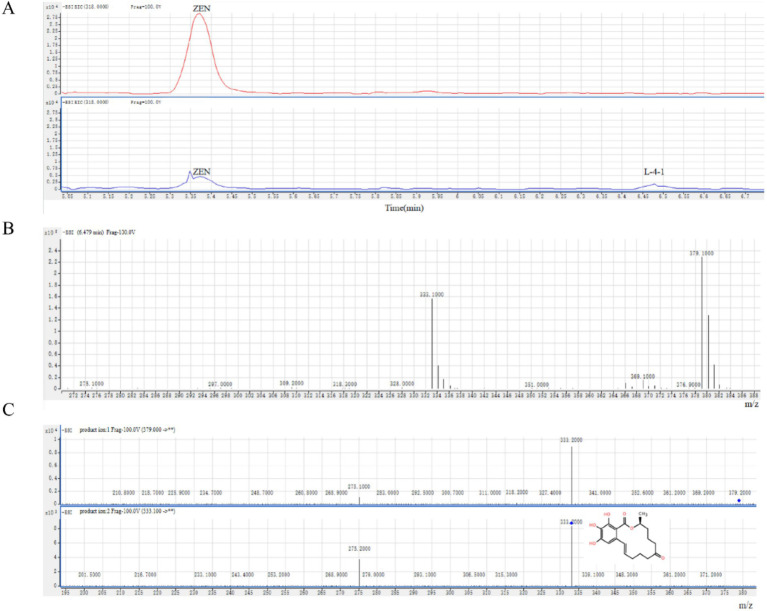
**(A)** ZEN degradation products produced by strain L-4. **(B)** Mass spectrometry and **(C)** secondary mass spectrometry analyses of the degradation product L-4-1.

By analyzing the degradation products, a potential degradation product was detected in the negative ion mode, which we designated L-4-1 (Figure 3A). L-4-1 was not detected in the liquid medium containing only ZEN, confirming that the substance was not derived from the liquid medium and ZEN. However, L-4-1 was found in the sample of L-4 bacterial solution +ZEN, indicating that L-4-1 was produced by L-4 degradation of ZEN. The results of mass spectrometry were shown in Figures 3B,C. The predicted molecular weight of L-4-1 was 333.1000 g/mol, which was consistent with the molecular formula C_18_H_23_O_6_ (OH-ZEN), but the ion fragment m/z201 was found in the secondary mass spectrometry analysis. Hildebrand et al. (2012) found that ionic fragments of m/z201 are present only in 15-OH-ZEN; therefore, we suggest that strain L-4 and the extracellular enzymes it produces can degrade ZEN to the product L-4-1 at low concentrations.

### 3.5 Detoxification effect of strain L-4 on ZEN-contaminated cornmeal

**Figure 4:**
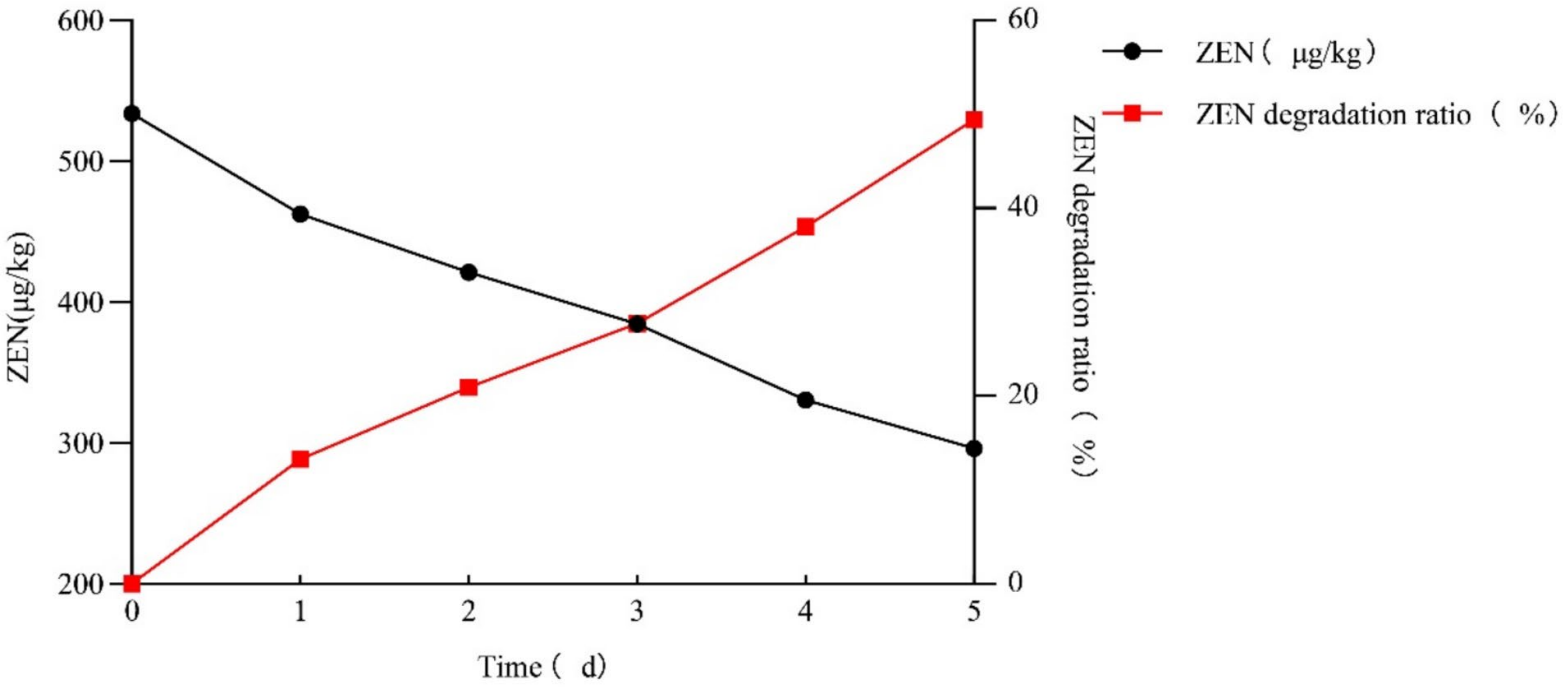
Rate of ZEN degradation by strain L-4 in cornmeal. Data are the mean ± SD of three independent replicates. As SD < 0.01, bar is not shown in the diagram.

As shown in Figure 4, the ZEN detoxification rate of strain L-4 against ZEN-contaminated cornmeal reached 49.41%. After 5 days of incubation, the ZEN concentration in cornmeal decreased from 532.91 μg/kg to 269.60 μg/kg.

### 3.6 Whole-genome analysis of strain L-4

#### 3.6.1 Basic genome annotation analysis

**Figure 5:**
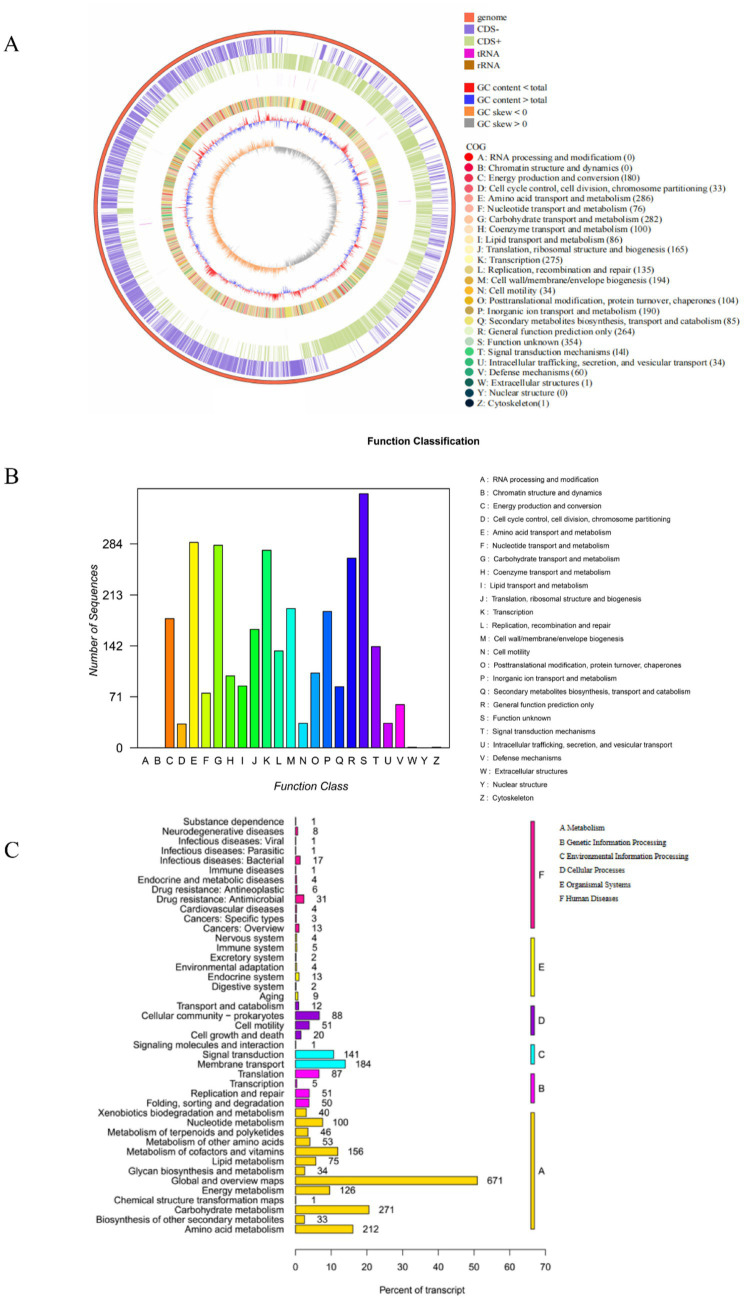
**(A)** *Bacillus mojavensis* L-4 sequencing gene loop diagram. **(B,C)** Functional annotations of the L-4 genome against COG and KEGG databases.

As shown in Figure 5A, the total length of the L-4 genome is 4,027,871 bp, with a GC content of 43.78% and 3,659 predicted protein-coding genes (CDS).

(1) COG annotation.

Functional classification and prediction of the genome of strain L-4 were performed using COG to infer protein functions. As shown in Figure 5B, 3,080 genes were annotated into 22 COG categories. The most abundant functional genes were “Function unknown (354 CDSs),” “Amino acid transport and metabolism (286 CDSs),” “Carbohydrate transport and metabolism (282 CDSs),” “Transcription (275 CDSs),” “Cell wall/membrane/envelope biogenesis (194 CDSs),” and “Inorganic ion transport and metabolism (190 CDSs).”

(2) KEGG metabolic pathway analysis.

Whole-genome sequencing of strain L-4 facilitated the investigation of the ZEN degradation mechanism. Figure 5C showed the KEGG annotation classification statistics of the L-4 genome, revealing 2,637 annotated genes. Most of the genes in strain L-4 were found to be related to metabolism, with 558 genes involved in carbohydrate, amino acid, and lipid metabolism. In particular, amino acid and lipid metabolism (such as *β*-oxidation of fatty acids) may be associated with the formation of hydroxyl groups in ZEN degradation products. Moreover, strain L-4 was noted to possess complete pathways for glycolysis, transmembrane transport, oxidative phosphorylation, tricarboxylic acid cycle, and pyruvate metabolism, indicating its robust metabolic capabilities conducive to ZEN degradation.

#### 3.6.2 Analysis of degradation-related features in genome annotation

(1) Genes associated with ZEN degradation.

**Figure 6:**
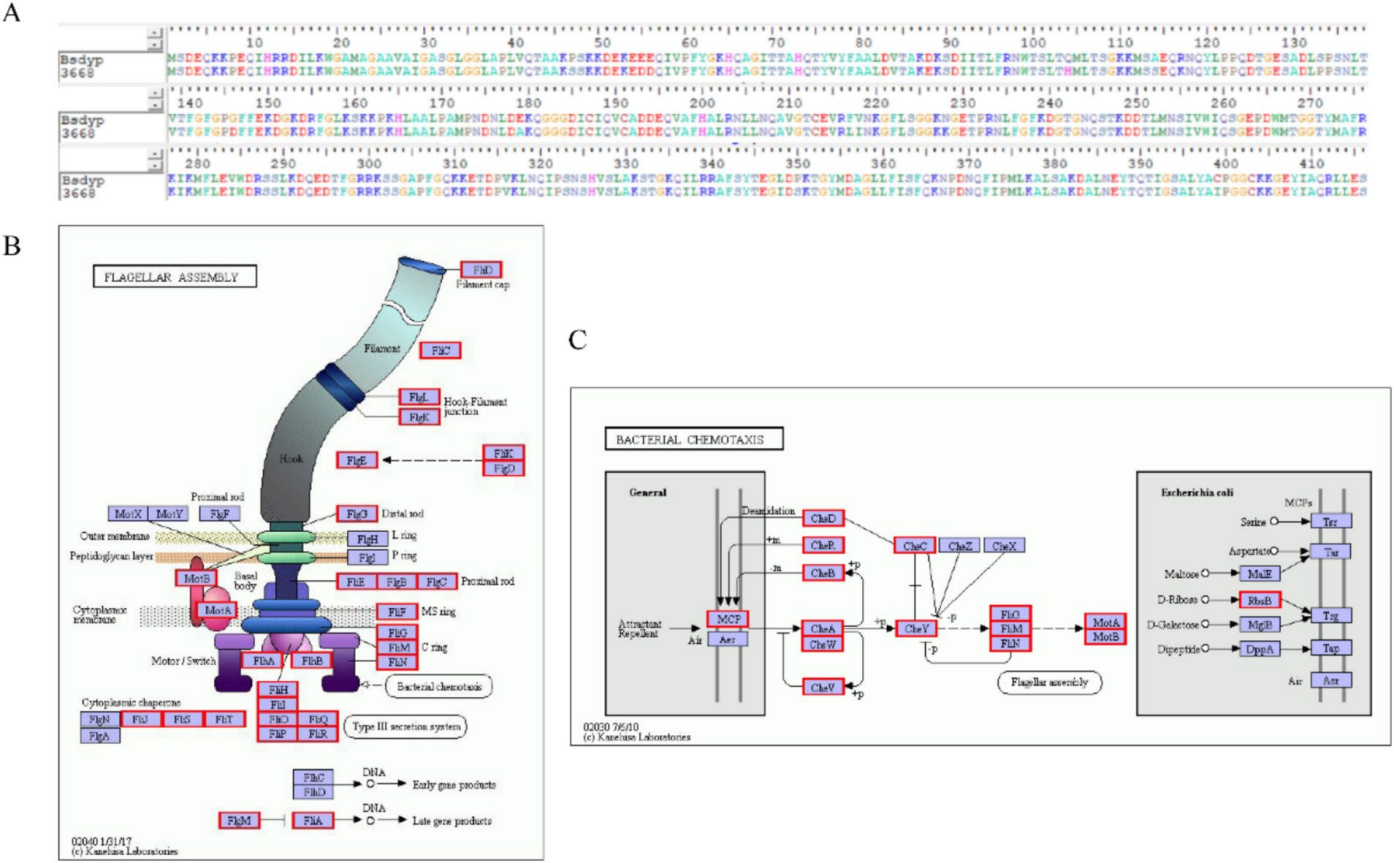
**(A)** Comparison of the protein sequence efeB 3,668 of *B. mojavensis* L-4 with the sequence of ZEN degradation protein BsDyP. **(B)** Flagellar assembly gene. **(C)** Annotation of bacterial chemotaxis genes in the L-4 genome.

Identifying enzymes involved in ZEN degradation is crucial for understanding the environmental transformation of ZEN and management of the degradation processes. As shown in Figure 6A, comparison of the 3,659 protein sequences annotated in strain L-4 with known ZEN-degrading enzyme sequences using DNAMAN software revealed a protein sequence with 95.91% homology to BsDyP from *Bacillus subtilis*.

(2) Flagella formation and chemotaxis.

Bacterial chemotaxis, encompassing flagellar motility and signal transduction, is essential for strain L-4 to navigate toward favorable conditions, enhancing ZEN degradation and bioavailability. As depicted in Figures 6B,C, strain L-4 exhibited complete flagellar assembly and chemotaxis-related genes.

#### 3.6.3 Subcellular localization, transmembrane region, signal peptide, hydrophobicity, and secondary structure of efeB 3,668

**Figure 7:**
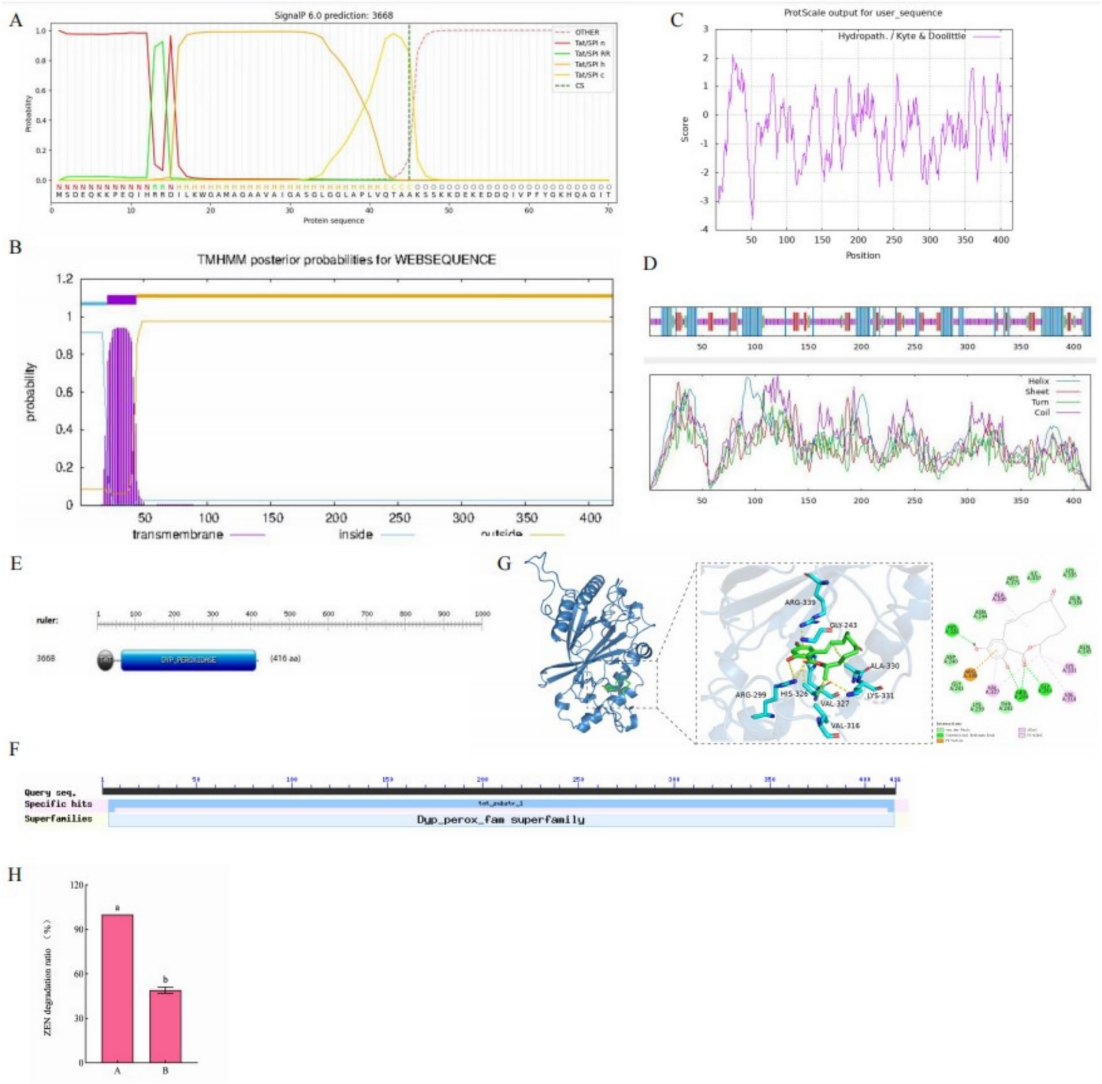
**(A)** Signal peptide, **(B)** transmembrane sequence, **(C)** hydrophilicity, **(D)** convoluted helical domain, **(E)** active center, and **(F)** molecular docking model characterizing the binding of ZEN to efeB 3,668. **(G)** Conserved structural domains of efeB 3,668. **(H)** Effect of catalase on ZEN degradation rate. A: no catalase was added to the supernatant; B: catalase was added to the supernatant.

By using PSORT, efeB 3,668 was predicted to primarily exist in the cytoplasm, presenting the following distribution: cyto (22.5%), cyto_nucl (20%), nucl (12.5%), mito (12.5%), plas (10%), extr (7.5%), cysk (7.5%), pero (5%), and E.R. (2.5%). SignalP 6.0 analysis of the amino acid sequence of efeB 3,668 (Figure 7A) identified a signal peptide. Furthermore, TMHMM 2.0 predicted a transmembrane sequence in efeB 3,668 (Figure 7B), suggesting its role as a transporter protein. ProtScale analysis showed Ala at position 25 with the highest hydrophobic value of 2.133 and Lys at position 53 with the maximum hydrophilic value of −3.633 (Figure 7C). In particular, the number of hydrophilic amino acid residues significantly exceeds that of hydrophobic amino acid residues throughout the entire polypeptide chain, suggesting that efeB 3,668 was a hydrophilic protein.

SOPMA predicted that the secondary structure of efeB 3,668 consisted of 29.33% *α*-helix, 14.42% extended strand, 5.29% *β*-turn, and 50.96% random coil (Figure 7D). ScanProsite software predicted the presence of two active centers in efeB 3,668: a TAT active site spanning amino acids 1–44 and a dyp_peroxidase active site spanning amino acids 63–408 (Figure 7E). NCBI analysis revealed two conserved domains in efeB 3,668: the TAT small family and Dyp_perox superfamily (Figure 7G).

ProtParam software analysis demonstrated the molecular weight of the protein to be approximately 45 kD, with a molecular formula of C_2024_H_3192_N_556_O_618_S_15_. The theoretical isoelectric point was 8.86, indicating alkaline characteristic. The number of negatively charged amino acid residues (Asp+Glu) was 48, while that of positively charged amino acid residues (Arg + Lys) was 54. The extinction coefficient was 6,405, instability index was 34.09, and the aliphatic index was 70.67.

#### 3.6.4 Molecular docking model representing the binding of efeB 3,668 (tertiary structure) to ZEN

By employing the 7pkx.1.A structure as a template, homology modeling of efeB 3,668 was conducted using SWISS-MODEL, which yielded a sequence identity of 95.91% and similarity score of 0.90. The functional prediction for efeB 3,668 was peroxidase. Subsequently, the 3D structure of efeB 3,668, obtained through homology modeling, was utilized to investigate its interaction with ZEN via molecular docking simulations. As shown in Figure 7F, the docking pattern revealed a binding energy of −6.9557 kcal/mol between ZEN and efeB 3,668, indicating a strong binding interaction. ZEN primarily interacted with efeB 3,668 through the formation of hydrogen bonds and hydrophobic forces. The hydrogen bonding interactions were observed between ZEN and residues His326, Arg299, and Gly243 on efeB 3,668, while hydrophobic interactions were noted between ZEN and residues Ala330, Lys331, Val316, and Val327 on efeB 3,668. In addition, the Arg339 residue on the receptor exhibited electrostatic interactions with ZEN. These findings collectively suggested that ZEN serves as a suitable substrate for efeB 3,668.

#### 3.6.5 Validation of the major ZEN degrading enzymes

When catalase was added to the supernatant, it was found that the degradation rate of ZEN was 49.84% at 72 h, which was lower than that of pure extracellular enzymes (as shown in Figure 7H). It is speculated that this is due to the reduction or absence of H_2_O_2_, but this confirms our previous inference that peroxidase plays a major role in the degradation of ZEN in L-4 bacterial solutions.

## 4 Discussion

In the present study, we investigated numerous bacterial and fungal strains for their ZEN degradation capabilities and identified a bacterial strain L-4 with excellent ZEN-degrading ability. Then, we conducted comprehensive taxonomic identification of strain L-4 utilizing diverse methodologies. The initial morphological observations provided preliminary insights into the identity of strain L-4. Subsequently, molecular biological identification methods, particularly 16S rDNA sequencing analysis and whole-genome sequencing, which demonstrated high sensitivity and specificity as well as remarkable advantages in species identification (Taş et al., 2021), confirmed the taxonomic affiliation of strain L-4 at the molecular level, ensuring the accuracy and reliability of our identification results. As shown in Figure 1, the morphological characteristics and sequencing results confirmed the classification of strain L-4 as *B. mojavensis*. Originally isolated from desert soil, *B. mojavensis* exhibited closest phylogenetic relationship with *B. amyloliquefaciens*, *B. atrophaeus*, and *B. subtilis* (Xu and Côté, 2003), suggesting that *B. mojavensis* could be considered as a subspecies of *B. subtilis*.

A variety of probiotics, including lactic acid bacteria, yeasts, and bacilli, have been observed to possess the ability to antagonize Fusarium and degrade ZEN. For instance, *S. cerevisiae* exhibited a 52% adsorption rate for 100.0 μg/mL ZEN (Chlebicz and Śliżewska, 2020), while *L. plantarum* 3QB361, a strain isolated from Brazilian cheese, achieved a 70–80% detoxification rate for 2.0 μg/mL ZEN within 15 min (Møller et al., 2021). Furthermore, *B. subtilis* YQ-1, isolated from soil, degraded 98.36% of ZEN in the fermentation broth (Zhou et al., 2024). However, unlike bacilli that can tolerate and survive in harsh environments and persist through processing and storage, while lactic acid bacteria are prone to deterioration during the processing, transportation and storage of food and feed, which limits their ZEN degradation efficiency. Moreover, yeasts are ineffective in detoxifying ZEN because they primarily adsorb ZEN onto their cell wall (Keller et al., 2015) and do not completely metabolize it. In the present study, the ZEN degradation ability of strain L-4 was studied under different conditions, and the results showed that strain L-4 exhibited excellent ZEN degradation ability in liquid medium and cornmeal. The degradation rate of 0.5 μg/mL ZEN by extracellular protein produced by strain L-4 in liquid medium reached 100%. Furthermore, strain L-4 showed remarkable adaptability to diverse conditions, wide pH and temperature range during fermentation. However, the ZEN degradation rate decreased as the pH of the medium and the culture temperature increased or decreased, which is consistent with the previous results on bacterial degradation of ZEN. Currently, there is a lack of research on the rapid and efficient degradation of concentrated ZEN in grains by semi-solid fermentation. We extended the incubation period to 72 h to ensure the complete removal of ZEN and take into account potential changes in bacterial activity under different experimental conditions. This method allowed us to confirm the stability and consistency of the degradation process over a longer period of time. In the present study, *B. mojavensis* L-4 achieved a 49.41% degradation rate of ZEN in cornmeal, outperforming other probiotics. Given its ability to degrade ZEN across a broad spectrum of concentrations, temperatures, and pH values, *B. mojavensis* emerged as a promising candidate for agricultural and industrial applications.

Despite significant progress in the analysis of key enzymes, degradation products, and mechanisms forn ZEN biodegradation, the potential unknown side effects or high levels of toxicity of ZEN and its derivatives continues to hinder the practical application of microbial ZEN degradation in industrial production (Tan et al., 2023). Although previous studies have demonstrated the effective degradation of ZEN by *B. licheniformis* CK1 (Fu et al., 2016), further analysis of their degradation products is scarce. During the degradation of ZEN, many derivatives were formed, including *α*-zearalanol with high estrogenic toxicity, as well as zearalenone 14-glucoside and zearalenone 14-sulfate with lower estrogenic toxicity. In the present study, the degradation product L-4-1 was analyzed using an anionic mode at m/z = 333.2000, similar to the report by Guo et al. (2024) who employed an anionic mode (m/z = 333.1331 [M-H]-) and estimated a relative molecular mass of 333 for the ZEN degradation product. However, the present study detected additional daughter ions at m/z 315.3000, m/z 306.5000, m/z 293.1000, m/z 279.0000, m/z 275.2000, and m/z 201.5000, which were generated by the extracellular proteins of strain L-4 during ZEN degradation. Sun et al. (2024) and Hildebrand et al. (2012) reported that the fragment ion at m/z 201 was exclusive to 15-OH-ZEN, indicating that this degradation product was a C15-hydroxylated metabolite of ZEN with low estrogenic activity (Drzymala et al., 2015). This finding is in agreement with the peroxidase BsDyP mediated effect observed by Qin et al. (2021), thus implying a molecular formula of C_18_H_23_O_6_ for the degradation product L-4-1.

The whole genome sequencing of strain L-4 provided valuable insights into the degradation mechanisms of ZEN and identification of related functional genes, and provides a theoretical basis for the study of bacterial enzyme-substrate interactions, metabolic pathways at the molecular level, and potential biological mechanisms. The genome circle plot of strain L-4 illustrated its genomic characteristics, encompassing gene distributions on both the forward and reverse strands, COG functional classification of genes, rRNA and tRNA distributions, as well as GC content, thereby facilitating a deeper understanding of its genetic information (Krzywinski et al., 2009). By utilizing Gene Ontology terminology (Ashburner et al., 2000), we annotated 3,956 genes within the L-4 genome. These genes were found to predominantly participate in a range of biological processes, including cellular metabolic processes, catalytic activity, binding capabilities, and transmembrane transport. In addition, in the cell component branch of strain L-4, the genes related to the overall composition of the cell membrane accounted for the largest proportion, followed by the organelles, reflecting the specific location of gene action. These data indicated that the protein functions of strain L-4 were primarily focused on cellular organization, catalysis, and metabolic processes, and had significant physiological effects on growth and ZEN biodegradation. The KEGG database is a comprehensive resource integrating genomic, chemical, and systems function information, and is the most extensively utilized database for metabolic pathways (Cao et al., 2019). In the KEGG analysis of the L-4 genome, a total of 2,637 genes were annotated, most of which are involved in metabolic processes. In a previous study by Qin et al. (2021) and Mwabulili et al. (2024) on *Bacillus* spp. using whole-genome sequencing, peroxidase and laccase enzymes were found to facilitate ZEN degradation.

We hypothesize that the degradation of ZEN by strain L-4 involves reduction and addition of relevant functional group bonds, as well as gene transcription and synthesis, which requires robust amino acid transport and metabolism. This highlights that strain L-4 requires vigorous metabolic activities and energy production, as evidenced by gene annotation (60 CDS) under the category “defense mechanism,” strain L-4 not only has vigorous growth and metabolic activities, but also has specific defense mechanisms and degradation capabilities. These results indicated that strain L-4 had a strong metabolic transformation ability during ZEN degradation. It must be noted that bacterial chemotaxis enhances ZEN degradation and bioavailability, and strain L-4 typically responds to environmental pollutants by degrading them. By utilizing pollutants as nutrients or energy sources, strain L-4 exhibits active chemotaxis (Parales et al., 2015). Moreover, bacterial chemotaxis encompasses flagellar motility and signal transduction, and strain L-4 possesses well-developed flagellar assembly and chemotaxis-related genes, facilitating its orientation toward favorable conditions.

The genome scanning and sequencing analysis of strain L-4 can help to understand the functional genes related to degradation, and provide a theoretical basis for studying bacterial enzyme-substrate interactions, metabolic pathways at the molecular level, and underlying biological mechanisms. By comparing the sequence of the degrading enzymes, we predicted that the protein sequence efeB 3,668 was involved in ZEN degradation. In a previous study, Ding et al. (2024) cloned the PoDyP4 enzyme and identified its ability to convert to 15-OH-ZEN detoxification through oxidation and hydroxylation, similar to the degradation products observed in the present study. Subsequently, we predicted the subcellular localization, transmembrane regions, signal peptides, hydrophobicity, and secondary and tertiary structures of efeB 3,668, as well as performed molecular docking with ZEN, which revealed that efeB 3,668 was an effective ZEN-degrading enzyme. Moreover, the degradation of ZEN by *B. mojavensis* L-4 in liquid cultures and cornmeal was found to be significantly influenced by environmental conditions, including temperature, pH, inoculum size, and ZEN concentration. Thus, identification of optimal conditions and prediction of degradation proteins can provide valuable insights for developing effective strategies for the control of ZEN in corn.

In conclusion, this study successfully isolated a ZEN-degrading *B. mojavensis* L-4 strain from chicken intestine. Under optimal conditions of pH 7.0 and 37°C, strain L-4 completely converted 0.5–1.0 μg/mL ZEN to 15-OH-ZEN, a metabolite with reduced estrogenic toxicity, within a 24-h period. Furthermore, *B. mojavensis* L-4 achieved a notable degradation rate of 49.41% for up to 532.91 μg/kg ZEN in cornmeal. The predicted peroxidase secreted by strain L-4 (efeB 3,668) was noted to be primarily responsible for ZEN detoxification through hydrogen bonding and hydrophobic interactions. These findings indicated that *B. mojavensis* L-4 and its peroxidase had significant application potential for mitigating ZEN contamination in food and feed.

## Data Availability

The 16S rDNA sequence of strain L-4 was submitted to the National Center for Biotechnology Information (NCBI) with GenBank ID 649301.1.

## Footnotes

^1^ http://pubchem.ncbi.nlm.nih.gov/
